# A Cross-Recurrence Analysis of the Pupil Size Fluctuations in Steady Scotopic Conditions

**DOI:** 10.3389/fnins.2019.00407

**Published:** 2019-04-30

**Authors:** Pietro Piu, Valeria Serchi, Francesca Rosini, Alessandra Rufa

**Affiliations:** ^1^Eye Tracking and Visual Application Lab, Department of Medicine, Surgery and Neuroscience, University of Siena, Siena, Italy; ^2^Neurology and Neurometabolic Unit, Department of Medicine, Surgery and Neuroscience, University of Siena, Siena, Italy

**Keywords:** pupil diameter, cross-recurrence quantification analysis, empirical mode decomposition, Epworth Sleepiness Scale, Gaussian-copula

## Abstract

Pupil size fluctuations during stationary scotopic conditions may convey information about the cortical state activity at rest. An important link between neuronal network state modulation and pupil fluctuations is the cholinergic and noradrenergic neuromodulatory tone, which is active at cortical level and in the peripheral terminals of the autonomic nervous system (ANS). This work aimed at studying the low- and high-frequency coupled oscillators in the autonomic spectrum (0–0.45 Hz) which, reportedly, drive the spontaneous pupillary fluctuations. To assess the interaction between the oscillators, we focused on the patterns of their trajectories in the phase-space. Firstly, the frequency spectrum of the pupil signal was determined by empirical mode decomposition. Secondly, cross-recurrence quantification analysis was used to unfold the non-linear dynamics. The global and local patterns of recurrence of the trajectories were estimated by two parameters: determinism and entropy. An elliptic region in the entropy-determinism plane (95% prediction area) yielded health-related values of entropy and determinism. We hypothesize that the data points inside the ellipse would likely represent balanced activity in the ANS. Interestingly, the Epworth Sleepiness Scale scores scaled up along with the entropy and determinism parameters. Although other non-linear methods like Short Time Fourier Transform and wavelets are usually applied for analyzing the pupillary oscillations, they rely on strong assumptions like the stationarity of the signal or the *a priori* knowledge of the shape of the single basis wave. Instead, the cross-recurrence analysis of the non-linear dynamics of the pupil size oscillations is an adaptable diagnostic tool for identifying the different weight of the autonomic nervous system components in the modulation of pupil size changes at rest in non-luminance conditions.

## Introduction

The pupil controls the amount of light radiations reaching the retina, by modulating its diameter through the interaction of two muscles under sympathetic-parasympathetic control. The pupil constriction is regulated by the contraction of the iris sphincter muscle receiving parasympathetic innervation mainly through cholinergic fibers. The pupil dilatation is instead related to the contraction of the radial muscle of the iris, under sympathetic control (Loewenfeld and Lowenstein, 1993). Due to the well-known neuroanatomical substrate, the clinical examination of the pupillary light reflex is considered an indicator of the optic nerve conduction, brainstem integrity, vigilance and coma. In recent years, studies in rodents and non-human primates found a tight coupling between pupil size and cortical state even during quiet wakefulness, suggesting a non-luminance mediated system for pupil size variations, associated to neural network oscillations. Studies combining electrophysiology, optical imaging and neural networks modeling, indicated that the link between brain state activity and pupil size is related to the neuro-modulatory effect of the noradrenergic and cholinergic systems (Murphy et al., 2014; Costa and Rudebeck, 2016; Joshi et al., 2016; Eckstein et al., 2017). In this respect, a direct relationship between pupil size and moment-to-moment fluctuations in the activity of noradrenergic neurons of the brainstem locus coeruleus (LC) has been verified (Aston-Jones and Cohen, 2005; Nassar et al., 2012). Other forebrain nuclei and cortical areas connected to LC are activated during spontaneous and event driven pupil size changes (Wang and Munoz, 2015; Joshi et al., 2016) suggesting a circuit for pupil response, linked to arousal, attention and perception systems (Jones, 2004; Naber et al., 2013; Wang and Munoz, 2015; Fazlali et al., 2016; Reimer et al., 2016; Larsen and Waters, 2018). Overall, these studies outline a new role for the pupil size monitoring as a reliable and non-invasive peripheral marker of rapid brain state changes (Hartmann and Fischer, 2014; Schwalm and Jubal, 2017).

From a methodological point of view, a challenge in the analysis of the pupil size variations is the identification of specific patterns that may be representative of changes in the cortical state activity. Different methods have been proposed to assess the pupillary spontaneous oscillations in isoluminant–non-accommodation inducing conditions or in the dark (Lüdtke et al., 1998; Pong and Fuchs, 2000; Zénon et al., 2014; Zénon, 2017). According to the assumptions those methods meet, we distinguish: stationary and linear assumption meeting methods, non-linear assumption meeting methods and non-linear and non-stationary assumption meeting methods. Like other physiological non-stationary signals, under steady stimulation, the pupillary oscillatory signal is expected to show non-linear and chaotic patterns (Poon and Merrill, 1997; Morad et al., 2000; Wilhelm et al., 2001; Merritt et al., 2004; Muppidi et al., 2013; Regen et al., 2013). The non-linear methods assume that the dynamics of the pupil size follow the rules of deterministic chaos rather than a stochastic or linear process (Rosenberg and Kroll, 1999). Common non-linear methods for the analysis of pupillary oscillations imply the use of the Short Time Fourier Transform (Nowak et al., 2008) and wavelets transformations (Henson and Emuh, 2010; Nowak et al., 2013; Reiner and Gelfeld, 2014). These methods assume an underlying stationary signal or require an *a priori* knowledge of the shape of the single basis wave; assumptions that do not well reflect the pupillary dynamics (Onorati et al., 2016). Among the most recent proposed non-linear and non-stationary meeting methods for the analysis of the pupil oscillations, there are the Hilbert Huang Transform, the EMD (Ruiz-Pinales et al., 2016; Villalobos-Castaldi et al., 2016), and the recurrence plots (Mesin et al., 2013, 2014; Monaco et al., 2014). The Hilbert-Huang transform is a frequency domain transformation, with the advantage of maintaining a good temporal and frequency resolution. Through the EMD, the original signal is split into components with slowly varying amplitude and phase, also known as IMFs. By applying a Hilbert transform to the IMF, instantaneous frequencies are generated as functions of time that give sharp identifications of embedded structures (Barnhart, 2011; Ruiz-Pinales et al., 2016; Villalobos-Castaldi et al., 2016). The RQA consists in taking single physiological measurements, projecting them into multidimensional space by embedding procedures and in identifying correlations that are not apparent in one-dimensional time series. This method provides quantitative indexes related to the number and duration of recurrences of the trajectory of a dynamical system in the phase space (Marwan, 2008; Webber and Marwan, 2015). Then, by applying the cross-recurrence analysis (CRQA) which is a bivariate extension of the RQA, we can investigate the dynamic interactions among the systems modulating pupil size oscillations. The use of CRQA has the advantage to better capture the recurring properties of a dynamic system given by the interaction over time of streams of information (Marwan, 2008; Coco and Dale, 2014). For this purpose, the EMD and CRQA were applied in succession. The main goal of our analysis was the identification of specific frequency components of the oscillatory signal comprised in the range of ANS, that could be quantified by couples of DET and ENT lying within the 95% prediction ellipse. Our result suggests that, in awake healthy subjects at rest, pupils oscillate in darkness with high frequency (HF) and low frequency (LF) components that are in the range of ANS, suggesting a balance between noradrenergic/cholinergic tone. Moreover, the position of the points on the ENT-DET plane seems to be related to the ESS score, and therefore, could give insights into the sleepiness state.

## Materials and Methods

### Participants

Twenty-six healthy subjects participated to the study (average age 36 ± 13 years old). The participants did not have neurological deficits or serious refractive problems. Moreover, the participants did not assume caffeine in the 2 h preceding the data collection (Wilhelm et al., 2014), and they reported to have slept more than 6 h in the night before the recording (average sleep hours 7.2 ± 0.1). The data collection was performed always between 3 and 6 pm. All subjects gave their written informed consent and the study respected the Declaration of Helsinki and was approved by the local Ethics Committee (Comitato Etico Locale Azienda Ospedaliera Universitaria Senese, EVAlab protocol CEL no. 48/2010).

### Experimental Setting

Pupil diameter recordings were performed monocularly with an ASL 504 eye-tracker device (Applied Science Laboratories, Bedford, MA, United States) sampling at a mean frequency of 240 Hz. The remote eye-tracker was placed at 650 mm far from the eye of the participant. The relative position of the subject’s head with respect to the eye-tracker was kept still by mean of a chinrest.

### Acquisition Protocol

Prior the data collection, the subjects were administered with an test ESS to investigate their vigilance state. ESS scores less than 11 are normally associated to subjects having normal sleepy state, while ESS scores greater than 11 suggested excessive daytime sleepiness (Parkes et al., 1998). ESS is a common used self-assessment questionnaire for the tiredness evaluation, hence it can turn to be a bias-prone measurement.

All the recordings were performed in a quiet light-controlled environment. To avoid the stimulation of pupillary light reflex, the subject was instructed to look straight for 15 min in a complete dark room (0 lux), similarly to the procedure adopted by Lüdtke et al. (1998). To reduce mental activity and cognitive load, subjects were instructed to try not to think to anything and to relax.

### Data Processing

**Figure 1:**
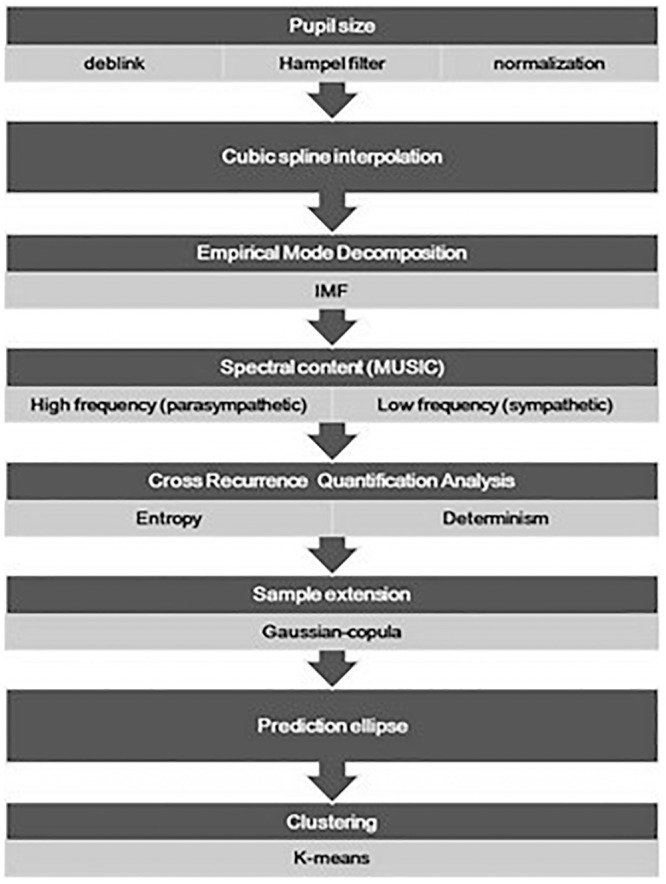
The flow chart presents the major procedures adopted for the analysis of the pupil size oscillation, from the data pre-treatment (deblink and artifact removal) and normalization, to the final drawing of the prediction ellipse. Data points of the prediction region in the entropy-determination plane underwent a further classification analysis and a pairwise comparison of the identified clusters was also done.

The flow chart of procedure employed to analyze the pupillary frequency balancing between the sympathetic and parasympathetic systems is shown in Figure 1. The pupil diameter data was exported in comma separated values format files and analyzed offline through Matlab (The Mathworks). The signal was de-blinked. Signal instances with the pupil diameter equal to zero were marked as blinks and removed from the signal. The remaining signal was then linearly interpolated. Moreover, machine artifacts introduced by the eye-tracker device due to failures to detect the pupil, were removed using Hampel filtering and low-pass filtered with a cut-off frequency (*f_0_*) of 2 Hz. The Hampel function computes the median of the data within moving windows. The width of the filter window (*w*) was determined accordingly to the ratio of the sample frequency (*f_s_*) over the cut-off frequency *f_0_* (Equation 1):

$$\text{w}\;=\;\text{0}.\text{44}\;\cdot \;{\text{f}}_{\text{s}}{\text{/f}}_{\text{0}}\;\;\;\;\;\;\;\;\;\;\;\;(1)$$

**Figure 2:**
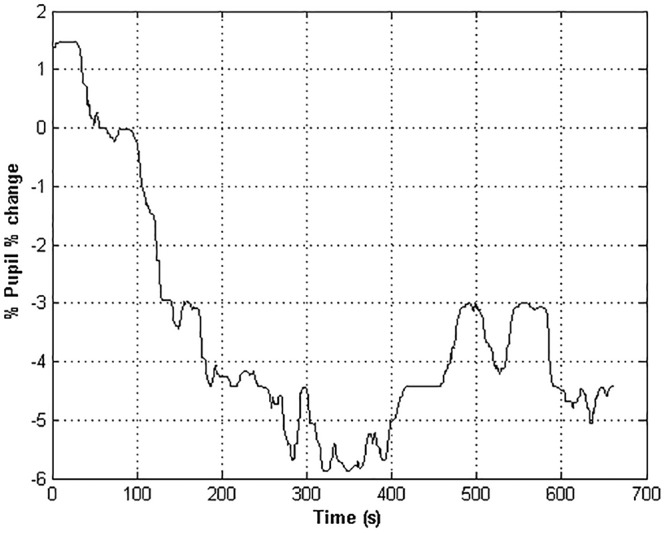
The time course of the pupil % change of an healthy subject. Negative values indicate a restriction of the pupil size with respect to the baseline value.

The variation of pupil size was computed with respect to a baseline value of the pupil estimated for each participant. Specifically, the baseline value of the pupil diameter signal was determined as the maximum value of the pupil size attained in the first 60 s of the signal in darkness (baseline), when the signal was expected to be more stable. The mean or the median of the pupil size were possible alternative reference values. However, taking the maximum value as reference enabled us to normalize the signal on the basis of a really observed value and to preserve the dynamics of the phenomenon. A baseline-corrected pupil diameter time series was then calculated as the diameter percentage change with respect to the value gathered in the basal condition (Equation 2).

$$\;\%change\;=\;\frac{X_{t}\;-\;Baseline}{Baseline}\cdot \;\;100\;\;\;\;\;\;\;\;\;\;\;\;\;\;\;(2)$$

where *X_t_* is the pupil diameter recorded at time *t*. The baseline correction provided the removal of inter-subject variability in pupil size percentage change of the pupil diameter signal (Lowenstein et al., 1963; Figure 2).

### Data Analysis

**Figure 3:**
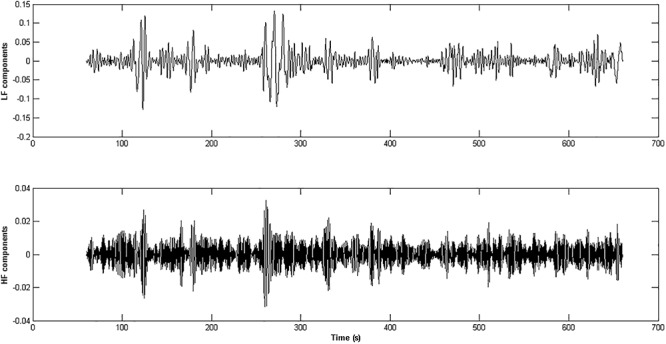
The panels represent the HF and LF components extracted from the pupil % change time series of an healthy subject. The IMFs obtained through the application of the EMD technique whose frequency content was inside the range [0.15 – 0.45 Hz] were aggregated and form the HF component of the ANS activity (lower panel), while the IMFs in the range [0 – 0.15 Hz] gave rise to the LF component (upper panel).

A cubic spline interpolation was used for compressing the percentage change time series with a resolution of five data points per second, which satisfied the Nyquist criterion (for the given 2 Hz cut-off frequency). The EMD was applied to the cubic spline interpolation of the percentage change time series in the autonomic frequency band ranging from 0 to 0.45 Hz (Huang et al., 1998). Since we were interested in a global spectral characterization of the IMFs derived from the EMD, the spectral content of the IMFs was estimated through MUSIC algorithms (Schmidt, 1986). The IMFs having most of the power in the autonomic frequency band were retained. The IMFs were then aggregated accordingly to the HF (0.15–0.45 Hz) and LF (0–0.15 Hz) ranges related to the parasympathetic and sympathetic systems activity (Cabrerizo et al., 2014; Figure 3). A CRQA was performed (Marwan and Kurths, 2002; Marwan, 2016) to assess the similarity between the dynamics of the parasympathetic and sympathetic processes by comparing the interaction between the LF and the HF components in the phase space. Three hyper-parameters must be set in the CRQA: EmbDim, TD, and the neighborhood radius (R). A symplectic geometry-based algorithm was used for estimating the EmbDim (Lei et al., 2002). The TD value was chosen as the one within the range (0: w/EmbDim) that maximized the sample entropy of the percentage change of pupil size. A FAN was taken as the neighborhood criterion, such that the cross-recurrence point density had a fixed predetermined value of 20%.

Two main parameters from CRQA were considered: the determinism (DET), which quantifies the fraction of periodic structures in the trajectories of the LF and HF dynamics in the phase space, and the entropy (ENT), which is the Shannon entropy of the diagonal line length distribution. Periodic signals are expected to yield high values of DET and small values of ENT (Marwan et al., 2007). To enlarge the sample size enough to apply clustering procedures on the DET-ENT plane and to investigate more carefully for possible highlights of this method on the analysis of the balancing of the sympathetic and parasympathetic systems thorough the analysis of the oscillations of the pupil diameter, we employed the Gaussian-copula simulation approach. Hence, firstly the association among age, ESS, ENT, and DET was measured by the Pearson’s correlation matrix. Then, a Gaussian-copula which maintained the dependence structure was used to generate one-hundred correlated multivariate data of those variables.

The percentage of pupil size change of each of the simulated data was then represented as a point on the DET-ENT plane.

### Statistical Analysis

On the simulated dataset the Doornik-Hansen multivariate normality test (Doornik and Hansen, 2008) was performed to verify the null hypothesis that the points in the DET-ENT plane were generated from a bivariate Gaussian distribution. The 95% prediction ellipse was calculated around the mean of observed points in the DET-ENT plane. Equations 3–4 indicate the formula for determining the length of the two semi-axes:

$$a_{x}\;=\;2\;\cdot \;\;\sqrt[{2}]{{\text{λ}}_{2}\cdot \frac{\left(n_{obs}\;-\;1\right)\;\cdot \;n_{var}\;\cdot \;f\;\left(1\;-\;\alpha ,\;\;n_{var},\;\;n_{obs}\;-\;n_{var}\right)}{n_{obs}\;-\;n_{var}}}\;\;\;\;\;\;\;\;\;\;\;\;\;\;\;\;\;\;\;\;\;\;\;\;\;(3)$$

$$a_{y}\;=\;2\;\cdot \;\sqrt[{2}]{{\text{λ}}_{2}\;\cdot \;\frac{\left(n_{obs}\;-\;1\right)\;\cdot \;n_{var}\;\cdot \;f\;\left(1\;-\;\alpha ,\;\;n_{var},\;\;n_{obs}\;-\;n_{var}\right)}{n_{obs}\;-\;n_{var}}}\;\;\;\;\;\;\;\;\;\;\;\;\;\;\;\;\;\;\;\;(4)$$

where *a_x_* and *a_y_* are the major and minor semi-axes of the ellipse, n_var_ is the number of variables (=2), *n_obs_* is the number of the observations (=100), λ_1_ and λ_2_ are the eigenvalues (in descending order) obtained from the spectral decomposition of the covariance matrix of ENT and DET, *f* is the pdf of the F distribution for the given significance *α* level and degrees of freedom (*n_var_*, *n_obs_-n_var_*). The orientation of the ellipse is given (in radians) by the direction of the eigenvector associated to the largest eigenvalue:

$$\text{θ}\;=\;atan\left(\frac{v_{y}}{v_{x}}\right)\;\;\;\;\;\;\;\;\;\;\;\;\;\;\;\;\;\;\;(5)$$

where *atan* is the inverse tangent function, and *v_x_* and *v_y_* are the components of the eigenvector corresponding to the largest eigenvalue. The coordinates of the points [*P_x_*, *P_y_*] laying on the ellipse contour are calculated as follows:

$$P_{x}\;=\;x_{c}\;+\;\left[\frac{a_{x}}{2}\;\cdot \;cos\;\left(t\right)\;\cdot \;cos\;\left(\text{ϑ}\right)\;-\;\frac{a_{y}}{2}\;\cdot \;sin\;\left(t\right)\;\cdot \;sin\;\left(\text{ϑ}\right)\right]\;\;\;\;\;\;\;\;\;\;\;\;\;\;\;\;\;\;\;\;\;\;\;\;\;(6)$$

$$P_{y}\;=\;y_{c}\;+\;\left[\frac{a_{x}}{2}\;\cdot \;cos\;\left(t\right)\;\cdot \;sin\;\left(\text{ϑ}\right)\;+\;\frac{a_{y}}{2}\;\cdot \;sin\;\left(t\right)\;\cdot \;cos\;\left(\text{ϑ}\right)\right]\;\;\;\;\;\;\;\;\;\;\;\;\;\;\;\;\;\;\;\;\;\;\;(7)$$

where *x_c_* and *y_c_* are the coordinates of the center of the ellipse, and *t* ranges in the interval [0, 2π].

The prediction region can provide the regulatory reference points for assessing if the underlying slow oscillations in the autonomic band of a new observed pupil size time series have the characteristics of a normal pattern.

Afterward, unsupervised clustering through K-means method with two clusters and a L1-norm distance function was applied within the elliptic prediction area. The two clusters were compared in covariance matrices and means vectors. Accordingly, the Box’s *M*-test was considered for verifying the homogeneity of the covariance matrices, and the Hotelling’s *T*^2^ test was used for testing the means. The variables age, ESS and *% change* associated to each cluster were separately compared through the Mann-Whitney unpaired test.

All statistical tests were two-sided and performed on Matlab with a 5% level of significance.

## Results

Self-organized adaptive systems like the brain generate complex signals which are inherently non-linear and non-stationary. Furthermore, unstable, weak, and state-dependent phase-locking characterizes the coupling between the biological oscillators (Shockley et al., 2002). Since the couplings between biological signals could also be predominantly transient, the canonical techniques of signal analysis, which basically rely on the assumption of stationary signals, are not appropriate. More importantly, the autonomic control of the spontaneous pupil fluctuations is expected to have non-linear/chaotic dynamics which can be well explored by recurrence analysis methods, whose domain is in the phase-space trajectories (Mesin et al., 2013). For these reasons, we chose the cross-recurrence method to analyze the spectral components of the ANS activity controlling the pupil fluctuations.

The EMD method was applied to the time series of pupil size variations to extract the low and high frequency components of the signal, which were found in the range of the ANS band. In fact, the EMD procedure, which is known to deal with non-linear and non-stationary signals like the pupil size oscillations, is a data driven method that overcomes the limitation of basis function shape typical of the wavelet decomposition method (Gonalves et al., 2007). The CRQA was then performed over the high and low frequency components and two parameters, i.e., entropy (ENT) and determinism (DET), were retained as the major features which quantified the non-linear dynamics of the high- and LF coupled oscillators in the autonomic band.

In Table 1 the sampling distributions of age, ESS scores, ENT, DET and average pupil change are reported. The sample declared a normal level of diurnal drowsiness (ESS: mean = 7.3; *SD* = 3.7). Five subjects (four of age lower than 30, and one of age greater than 60) reported relatively high ESS scores (>10). The cross-recurrence analysis returned low values both for ENT (mean = 0.92; *SD* = 0.09) and for DET (mean = 45.28%; *SD* = 7.46%). The percentage of pupil change in the sample (*% change*) (mean = -0.16%; *SD* = 1.91%) indicated an overall slight loss of pupil size with respect to the baseline, but high variability of the fluctuations as well.

**Table 1:** Sampling distributions of age, Epworth Sleepiness Scale, entropy, determinism, and average pupil change.

Age	ESS	Entropy	% Determinism	% Change
24	3	1.08	52.34	-0.38
24	5	0.97	49.64	0.85
24	5	0.96	56.69	3.97
24	6	1.00	57.39	-2.36
24	13	1.03	46.94	-1.69
24	14	0.90	48.86	-2.00
25	6	0.86	37.91	-1.57
25	14	0.92	43.09	0.30
27	2	0.84	38.14	1.94
27	7	0.86	50.49	0.74
27	9	1.00	37.73	-3.35
28	3	0.89	41.85	-1.37
28	4	0.83	41.29	0.19
29	5	0.87	42.43	1.72
29	10	0.83	37.35	-1.37
29	13	0.97	56.99	1.39
31	5	0.72	29.79	1.43
33	8	0.83	44.9	-2.02
46	7	0.88	39.78	-2.60
46	9	0.99	46.22	-0.40
49	6	0.77	45.12	1.16
50	3	0.90	38.35	-3.46
51	4	0.95	35.6	0.33
56	7	1.00	51.47	2.34
62	14	0.94	56.19	2.07
63	8	1.04	50.69	-0.08

We firstly analyzed the possible association among the observed age, ESS, ENT, and DET. Based on the results, the DET and ENT variables were not significantly correlated to the age and the ESS score of the participants. Instead, a significant correlation between ENT and DET was found (*r* = 0.58, *p* = 0.002).

**Figure 4:**
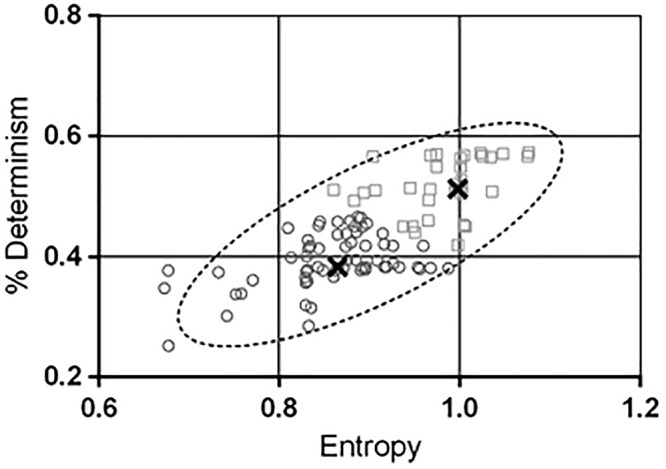
The contour of the ellipse envelopes the region in the entropy-determinism space where new measured combinations of entropy (ENT) and determinism (DET) will fall with a 95% probability. The null hypothesis that the two variables are generated from a bivariate normal distribution was verified through the Doornik-Hansen test. Values of DET in the y-axis have been rescaled in the range [0 – 1]. A K-means algorithm was used to characterize sub-areas within the prediction ellipse and two distinct clusters were found out. The thick black crosses indicate the centres of the clusters. Cluster 2 (squares) exhibited significant higher combination of ENT and DET than cluster 1 (circles). In addition the ESS resulted greater in cluster 2 compared to cluster 1. This finding suggested that the ENT-DET bivariate distribution inherently conveys information about the drowsiness level of the subjects.

The bivariate distribution of ENT and DET obtained from Gaussian-copula simulated points (Table 2) is depicted in Figure 4, together with the 95% prediction ellipse. The simulated values of ENT (mean = 0.90; *SD* = 0.09) and DET (mean = 43.66%; *SD* = 7.43%) were consistent with the values observed in the sample.

**Table 2:** Values of age, Epworth Sleepiness Scale, entropy, determinism, and average pupil change generated from a Gaussian-copula.

Age	ESS	Entropy	% Determinism	% Change	Age	ESS	Entropy	% Determinism	% Change
24	1	0.76	33.90	-2.02	29	5	0.83	31.97	-1.37
24	7	0.91	51.04	-1.94	29	5	1.00	51.30	0.29
24	11	0.86	43.69	-1.37	29	6	0.96	37.97	-0.40
24	9	0.90	41.85	-1.37	29	4	0.89	37.76	-1.75
24	10	0.84	45.84	-3.04	29	6	0.91	43.87	-1.37
24	6	0.95	38.26	-3.55	29	7	0.83	41.42	-3.45
24	4	0.97	49.38	-1.37	29	3	0.88	42.35	0.42
24	5	0.93	41.80	-3.42	29	3	0.91	39.35	-2.02
24	8	0.89	44.10	-2.01	29	7	0.83	42.78	1.84
24	5	1.04	50.81	-2.01	29	5	0.87	38.07	0.48
24	9	0.84	41.36	-1.92	30	4	0.86	37.73	0.76
24	3	0.92	38.32	-3.42	31	6	0.83	36.60	-0.39
24	6	0.99	50.99	-1.37	31	14	0.97	56.78	2.23
24	14	0.81	39.87	-3.47	32	9	0.94	45.02	-0.39
24	4	0.83	35.69	-3.44	32	14	0.84	45.25	-1.55
24	3	0.77	36.14	-2.54	34	8	0.96	45.95	-0.39
24	14	0.97	51.16	-2.49	36	4	0.85	37.75	-3.36
24	12	0.97	57.02	-1.98	41	13	0.86	51.06	0.30
24	5	1.00	56.28	-1.37	41	6	0.83	35.96	-1.37
24	5	1.03	56.53	0.20	41	5	0.87	43.81	1.34
24	13	1.00	51.29	-2.28	42	2	0.90	38.30	2.15
24	5	1.00	50.54	1.71	43	6	0.89	46.47	1.52
25	5	0.87	41.70	-1.87	46	6	0.94	51.45	1.63
25	14	1.08	57.34	-2.48	46	3	0.68	37.74	1.39
25	14	0.83	41.76	-1.42	46	3	1.00	45.27	0.06
25	8	0.90	37.86	-2.92	46	7	1.00	52.01	0.31
26	3	0.93	38.25	-1.38	46	14	1.03	56.75	1.93
27	9	0.97	54.93	-2.01	46	14	1.00	52.63	-1.47
27	4	0.74	30.17	-1.67	46	6	0.89	50.60	1.42
27	2	0.88	49.28	1.39	46	14	0.83	37.84	-1.75
27	6	0.81	44.77	-1.04	46	5	1.00	54.86	-1.64
27	4	0.86	36.67	-2.27	47	3	0.92	38.32	0.33
27	3	0.75	33.77	0.74	47	8	0.90	56.60	1.76
27	5	0.88	39.46	0.31	48	4	0.89	46.61	1.42
27	3	1.00	41.90	-0.43	49	3	0.83	37.95	1.41
27	3	0.83	37.81	0.71	49	9	0.95	45.01	-2.25
27	6	0.83	37.51	-3.07	49	9	0.83	40.07	0.47
28	3	0.86	45.83	2.08	50	3	0.99	38.08	-2.01
28	7	0.95	43.95	-1.75	50	3	0.84	31.53	0.84
28	3	0.93	38.87	0.08	50	7	1.00	56.82	1.96
28	5	0.84	38.25	1.05	51	7	0.88	45.02	2.03
28	3	0.88	39.49	2.02	51	13	0.97	38.15	1.47
28	3	0.67	34.75	-0.67	51	7	0.73	37.41	2.03
28	5	0.83	28.51	-2.04	52	3	0.89	44.98	1.96
28	3	0.90	45.54	1.56	53	8	1.08	56.66	2.05
28	8	0.87	38.23	-2.39	56	14	1.05	57.08	1.15
28	7	0.89	38.19	0.03	56	7	0.96	41.81	-0.09
29	3	0.68	25.23	-1.04	60	5	0.92	42.09	-1.57
29	9	0.88	45.97	0.19	61	5	1.01	44.96	1.08
29	12	1.00	50.96	1.70	63	7	1.02	57.21	2.28

The major parameters of the prediction ellipse are displayed in Table 3. The coordinates of the center of the ellipse are the means of the simulated ENT and DET vectors. The axes of the ellipse indicate the magnitude of the inertia along the directions of ENT and DET. The interval estimations of ENT and DET were obtained through Equations 6 and 7. Table 4 displays the expected intervals of determinism for equally spaced intervals (0.05 bits) of entropy.

**Table 3:** Parameters of the 95% prediction ellipse in the ENT-DET plane.

Center	(0.90, 0.44)
Semiaxis (x)	0.53
Semiaxis (y)	0.20
Angle (rad)	0.69
Angle (degree)	39°44’
Hyper-volume	0.08
Perimeter	0.12
Eccentricity	0.92

**Table 4:** Normative intervals of determinism by ranges of entropy.

Entropy	% Determinism
0.70–0.75	24–40
0.75–0.80	24–46
0.80–0.85	24–51
0.85–0.90	26–55
0.90–0.95	29–58
0.95–1.00	33–60
1.00–1.05	38–60
1.05–1.10	45–60

Through the K-means procedure, two clusters of points were identified within the prediction ellipse and their descriptive statistics is shown in Table 5.

**Table 5:** Descriptive statistics of the clusters identified within the prediction ellipse.

	Cluster 1		Cluster 2	
	Mean	SD	Mean	SD
Age	33.1	10.2	36.3	12.4
ESS	5.7	3.2	8.2	3.7
Entropy	0.86	0.07	0.98	0.05
% Determinism	39	4	52	4
% Change	-0.69	1.77	-0.12	1.64

The generated ENT-DET values underwent the Doornik-Hansen multinormality test. The hypothesis of bivariate normal distribution was not rejected (DH statistic = 6.97, *p* = 0.14).

The covariance matrices of the clusters were not significantly different (Box’s *M*-test = 3.2; *p* = 0.37). The result of the Hotelling *T*^2^ test indicated that the bivariate ENT-DET means vectors between the clusters were significantly different (*T*^2^ = 200.8; *p* < 0.0001). The two clusters exhibited also significant different ESS scores (*U*-test = 701.5; *p* = 0.002), whilst they did not differ in age (*U*-test = 1073; *p* = 0.64), nor in *% change* (*U*-test = 933.5; *p* = 0.14).

## Discussion

The analysis of the pupil size oscillations is a promising diagnostic tool, enabling improvements in the identification of cortical state changes. Variations of cortical state activity during wakefulness have a strong influence on neural, perceptual and behavioral responses. Pupil diameter varies not only in response to variation of luminance and accommodation, but also during changes in alertness, attention, mental effort and decision making, suggesting a direct link between pupil size variation and cortical state changes (Preuschoff et al., 2011; Nassar et al., 2012; Naber et al., 2013; Alnæs et al., 2014; de Gee et al., 2014). Changes in the cortical state are associated to well characterized variations of the cortical signal frequency. Specifically, in awake rodents the investigation of local field potentials demonstrated the prevalence of LF fluctuations during periods of quiet resting. However, the initiation of locomotion or whisking was related to the suppression of low frequency components and increased high frequency oscillations (Poulet et al., 2012; Eggermann et al., 2014, McGinley et al., 2015b). This transition between slow and fast cortical activity was also observed across cortical regions (Poulet and Crochet, 2019). Electrophysiological studies have revealed that pupil constriction is associated to slow and synchronous cortical responses and inattentive behavior. Conversely, the cortical activation during task engagement or locomotion shows a persistent desynchronized neuronal activity associated to the dilatation of the pupil (Reimer et al., 2014, 2016; McGinley et al., 2015b; Schwalm and Jubal, 2017). Pupil size fluctuations and cortical state variations are modulated by the central noradrenergic and cholinergic pathways. Thus, monitoring pupil dynamics could be a reliable proxy of the changes in cortical states (Reimer et al., 2014, 2016; McGinley et al., 2015a,b). More specifically, the release of acetylcholine (Ach) from the basal forebrain and noradrenaline (NA) from LC have been shown to drive both the state of cortical connectivity and the pattern of the pupil size oscillations also in resting conditions (Reimer et al., 2016; Schwalm and Jubal, 2017). At the peripheral level, both Ach and NA are neurotransmitters of the ANS (parasympathetic and sympathetic systems, respectively) also controlling the pupil diameter. Overall, these premises encourage exploring new and reliable techniques for pupil dynamics monitoring that allow the identification of parameters attributable to NA and Ach modulatory effect in various cortical state changes.

We propose here, a method that can be used as a quantitative measurement of the non-linear dynamics of the pupil fluctuations. We applied a cross-recurrence technique for estimating determinism (DET) and entropy (ENT) features and their distribution, in order to quantify the degree of coupling between the oscillators of the low (LF) and high frequency (HF) components of the pupillary signal. To the best of our knowledge this is the first study on the use of the ENT-DET plane for analyzing the dynamical systems associated to pupil size fluctuation during stationary scotopic visual conditions.

In our cohort of subjects, we observed low levels of determinism (<60%) and entropy (<1). This is consistent with spontaneous physiological signals recorded from healthy subjects, which are expected to be highly complex. Actually, low determinism can be associated to increase in the uncertainty of the signals, and hence to increase in the signal chaotic properties (i.e., complexity). In facts, complex systems are typically highly ordered. Therefore, they tend to preserve low entropy and counteract the second law of thermodynamics (*free energy principle*). A *de-complexification* process occurs when free-running physiological signals present sustained loss of complexity. The loss of complexity leads to less ordered states with higher entropy and with stronger coupling of the oscillators controlling the expression of the signal. This degradation in complexity is typically observed in pathological conditions or advanced aging. Therefore, the major result of this study is the identification of a normative elliptical region in the ENT-DET plane for the pupillary oscillators that could be compared with data from group of patients with neurodegenerative diseases. We hypothesize that the occurrence of points outside of the defined elliptical prediction region may signal potential pathological conditions related to alterations in the ANS. As secondary outcome, we observed that, within the elliptical region of confidence, clusters of points with different characteristics of ENT-DET highly differed also in their ESS scores. This finding suggests that the location of the points in the ENT-DET plane can also reveal alterations in the sleepiness state.

Our results indicate that in resting wakefulness conditions, without the influence of light and accommodation, pupil size oscillations are under the effect of a balanced cholinergic/noradrenergic tone. We believe that the employed CRQA-based method may help to lay the groundwork for studying the LF and HF components of the pupil, which may be related to neuronal network state of the brain at rest. Importantly, it consists in a non-invasive procedure that could be easily adopted in clinical context and for diagnostic assessment such as neurodegenerative conditions. Furthermore, this method is adaptable to different experimental conditions (e.g., variations of the visual stimulus, recording during cognitive tasks, etc) provided that the opportune frequency components are dug out from the signal. The joint recording of the pupil size fluctuations along with other physiological signals (e.g., heart rate variability, EEG, etc) would improve the method, since the study of possible time-dependent and/or frequency-related changes in autonomic functions would be facilitated by this integration.

## Ethics Statement

The study was approved by the local Ethical Committee Comitato Etico Locale Azienda Ospedaliera Universitaria Senese, EVAlab protocol CEL no. 48/2010.

## Author Contributions

All authors conceived and designed the study, critically revised the manuscript, and approved the final version of the manuscript. FR and VS acquired the data. AR, PP, and VS involved in the analysis and interpretation of data, and drafted the manuscript. AR revised the scientific content of the study.

## Conflict of Interest Statement

The authors declare that the research was conducted in the absence of any commercial or financial relationships that could be construed as a potential conflict of interest.

## Abbreviations

ANS: autonomic nervous system
CRQA: cross-recurrence quantification analysis
DET: percentage of determinism calculated from the cross-recurrence analysis
EmbDim: embedding dimension, a hyper-parameter to be estimated for the CRQA
EMD: empirical mode decomposition
ENT: entropy calculated from the cross-recurrence analysis
ESS: Epworth Sleepiness Scale
FAN: fixed amount of nearest neighbor
HF: high-frequency component in the ANS spectrum
IMFs: intrinsic mode functions extracted through the EMD
LF: low-frequency component in the ANS spectrum
MUSIC: multiple signal classification algorithm
R: neighborhood radius, a hyper-parameter to be estimated for the CRQA
RQA: recurrence quantification analysis
TD: time delay, a hyper-parameter to be estimated for the CRQA.
